# 

**Published:** 1895-11

**Authors:** 


### CONTENTS.

ORIGINAL COMMUNICATIONS—

OPENING ADDRESS AT THE SOUTHERN MEDICAL COLLEGE, OCTOCER 1ST. By Henry F. Harris, M.D., Atlanta, Ga. .... 513

THE SURGICAL TREATMENT OF CHOLELITHIASIS. By Hugh M. Taylor, M.D., Richmond, Va. .... 528

URIC ACID AS A FACTOR OF DISEASE. By E. Van Goidtsnoven, A.M., M.D., Atlanta, Ga. .... 535

SOCIETY REPORTS—

ATLANTA SOCIETY OF MEDICINE .... 541

SEVENTH ANNUAL MEETING OF THE TRI-STATE MEDICAL SOCIETY OF ALABAMA, GEORGIA AND TENNESSEE .... 548

EDITORIAL—

LOUIS PASTEUR .... 556

EXECUTION BY ELECTRICITY .... 557

DR. BATTEY .... 558

SELECTIONS AND ABSTRACTS—

EYE, EAR, NOSE AND THROAT .... 559

PRACTICAL CHEMISTRY AND PHARMACY .... 563

SURGERY .... 565

MEDICAL ITEMS .... 570

BOOK REVIEWS .... 572

ADVERTISERS' INDEX TO ADVERTISEMENTS .... xiv

MEDICAL ITEMS—CONTINUED .... x, xi, xii, xiii

CLASSIFIED INDEX TO ADVERTISEMENTS .... xxxiii

